# Retraction: Benchmarking Neuromorphic Systems with Nengo

**DOI:** 10.3389/fnins.2015.00460

**Published:** 2015-11-25

**Authors:** Neuroscience Editorial Office

The authors and the journal wish to retract the 19 October 2015 article cited above. The authors requested the retraction of this article in November 2015 as data was included in this article which had been released without the permission of all collaborators involved in the research project.

